# A Rho-associated coiled-coil containing kinases (ROCK) inhibitor, Y-27632, enhances adhesion, viability and differentiation of human term placenta-derived trophoblasts *in vitro*

**DOI:** 10.1371/journal.pone.0177994

**Published:** 2017-05-19

**Authors:** Kenichiro Motomura, Naoko Okada, Hideaki Morita, Mariko Hara, Masato Tamari, Keisuke Orimo, Go Matsuda, Ken-Ichi Imadome, Akio Matsuda, Takeshi Nagamatsu, Mikiya Fujieda, Haruhiko Sago, Hirohisa Saito, Kenji Matsumoto

**Affiliations:** 1 Department of Allergy and Clinical Immunology, National Research Institute for Child Health and Development, Tokyo, Japan; 2 Center of Maternal-Fetal, Neonatal and Reproductive Medicine, National Center for Child Health and Development, Tokyo, Japan; 3 Division of Advanced Medicine for Virus Infections, National Research Institute for Child Health and Development, Tokyo, Japan; 4 Department of Obstetrics and Gynecology, Faculty of Medicine, The University of Tokyo, Tokyo, Japan; 5 Department of Pediatrics, Kochi Medical School, Kochi, Japan; NCMLS, Radboud University Nijmegen Medical Center, NETHERLANDS

## Abstract

Although human term placenta-derived primary cytotrophoblasts (pCTBs) represent a good human syncytiotrophoblast (STB) model, *in vitro* culture of pCTBs is not always easily accomplished. Y-27632, a specific inhibitor of Rho-associated coiled-coil containing kinases (ROCK), reportedly prevented apoptosis and improved cell-to-substrate adhesion and culture stability of dissociated cultured human embryonic stem cells and human corneal endothelial cells. The Rho kinase pathway regulates various kinds of cell behavior, some of which are involved in pCTB adhesion and differentiation. In this study, we examined Y-27632’s potential for enhancing pCTB adhesion, viability and differentiation. pCTBs were isolated from term, uncomplicated placentas by trypsin–DNase I–Dispase II treatment and purified by HLA class I-positive cell depletion. Purified pCTBs were cultured on uncoated plates in the presence of epidermal growth factor (10 ng/ml) and various concentrations of Y-27632. pCTB adhesion to the plates was evaluated by phase-contrast imaging, viability was measured by WST-8 assay, and differentiation was evaluated by immunofluorescence staining, expression of fusogenic genes and hCG-β production. Ras-related C3 botulinum toxin substrate 1 (Rac1; one of the effector proteins of the Rho family) and protein kinase A (PKA) involvement was evaluated by using their specific inhibitors, NSC-23766 and H-89. We found that Y-27632 treatment significantly enhanced pCTB adhesion to plates, viability, cell-to-cell fusion and hCG-β production, but showed no effects on pCTB proliferation or apoptosis. Furthermore, NSC-23766 and H-89 each blocked the effects of Y-27632, suggesting that Y-27632 significantly enhanced pCTB differentiation via Rac1 and PKA activation. Our findings suggest that Rac1 and PKA may be interactively involved in CTB differentiation, and addition of Y-27632 to cultures may be an effective method for creating a stable culture model for studying CTB and STB biology *in vitro*.

## Introduction

The syncytiotrophoblast (STB), a large multinucleated syncytium, covers the surface of placental villi, is in direct contact with the maternal blood and contributes to unique placental functions such as gas exchange, nutrition and ion supply to the fetus, waste transfer, pregnancy-specific hormone production and the immunological barrier [1]. STB dysfunction leads directly to placental insufficiency and may cause small for gestational age, premature delivery, infertility or even fetal death. In order to study the pathogenesis of such disorders, *in vitro* experiments using human STB seem to provide invaluable results, presumably equivalent to what happens in human placentas.

A trophoblast cell line, BeWo, is the most popular STB model for placental research [2]. However, experiments using trophoblast cell lines have some limitations, since they merely fuse spontaneously [3], and their gene expression profile correlates weakly with that of CTBs [4–6].

A primary culture STB model has been used to overcome these limitations. Although it is impossible to culture placenta-derived STB, previous studies demonstrated that term human placenta-derived primary cytotrophoblasts (pCTBs), which are the progenitor of STB, can be obtained [7–9]. In contrast to trophoblast cell lines, pCTBs reportedly differentiate into STB spontaneously (i.e., fuse to form syncytia and produce STB-specific proteins and hormones such as human chorionic gonadotropin β subunit (hCG-β) and placental lactogen), and they have been suggested to be a good model for STB [7, 10, 11]. However, because of decreased adhesion ability, loss of proliferation [11] or some other unknown reason(s), *in vitro* culture of pCTB is not always easily accomplished. That is a serious obstacle for many researchers in the field of villous trophoblast biology. Therefore, there has been an urgent need to improve pCTB adhesion and differentiation *in vitro*.

Some primary human cells are also reportedly difficult to culture. Specifically, in dissociation culture of human embryonic stem cells (hESC) and human corneal endothelial cells (hCEC), it was reported that activation of Rho-associated coiled-coil containing protein kinase (ROCK; a downstream effector kinase of Rho) prevented cell adhesion to tissue culture plates and induced apoptosis. Y-27632, a specific inhibitor of ROCK, inhibited the apoptosis and improved both cell adhesion to the substrate and culture stability [12–15]. Rho family proteins and their effectors are ubiquitously distributed and regulate various cell behaviors, including cell migration, cell adhesion to substrate, cell-to-cell fusion and apoptosis [16–18]. Moreover, previous studies found that changes in cellular behavior and activity occurred during differentiation of CTBs, including cytoskeletal and focal adhesion rearrangement [19, 20]. Therefore, we hypothesized that Rho family proteins are also involved in pCTB adhesion and differentiation, and that a ROCK inhibitor would enhance those events in pCTBs in the same manner as reported in hESC and hCEC. To test this hypothesis, we examined the effects of a ROCK inhibitor, Y-27632, on pCTB adhesion, viability and differentiation *in vitro*.

## Materials and methods

### Trophoblast isolation from term placentas

All human placentas used in this study were obtained from The Center for Maternal-Fetal, Neonatal and Reproductive Medicine of the National Center for Child Health and Development (NCCHD, Tokyo, Japan) after receiving written informed consent from each subject. This study was approved by the Ethics Committee of the National Center for Child Health and Development (No. 476).

pCTBs were isolated from term uncomplicated placentas (delivered by scheduled cesarean section) using a trypsin-DNase I, Percoll gradient centrifugation method [7–9], with slight modification.

Briefly, placental villous tissues (approximately 60 g) were washed with sterile saline, minced finely with scissors and washed again. The tissues were transferred into an enzyme digestion buffer (HBSS with 25 mM HEPES without Ca^2+^ or Mg^2+^, containing 0.25% trypsin (Gibco BRL; Gaithersburg, MD), 300 U/ml DNase I (Roche; Basel, Switzerland) and 2.4 U/ml Dispase II (Roche)) and incubated in a 37.0°C water bath with agitation (rotating at 150 rpm) for 20 minutes. This enzyme digestion was repeated three times, the supernatants were collected, and the enzyme activities were neutralized with fetal bovine serum (FBS; Biological Industries; Beit Haemek, Israel). The cells were resuspended in Iscove’s modified Dulbecco’s medium (IMDM; Meditech; Herndon, VA) supplemented with 10% FBS, 2 mM L-glutamine (Life Technologies; Grand Island, NY) and 100 IU/ml penicillin/streptomycin/amphotericin B (Wako Pure Chemical Industries; Tokyo, Japan) (cell-culture IMDM). The cells were filtered through a 100-μm nylon cell strainer (Falcon; Durham, NC), resuspended in HBSS, layered on discontinuous (50%:45%:25%:20%) Percoll (GE Healthcare; Piscataway, NJ) gradients, and centrifuged at 1,200 g for 20 minutes. Cells at the 45% Percoll layer were collected and washed with cell-culture IMDM. The cells were counted, suspended in Cellbanker (ZENOAQ; Fukushima, Japan) and stored in a liquid nitrogen freezer until use.

Contaminating cells such as placental fibroblasts, leukocytes and extravillous trophoblasts were depleted right after thawing by using a negative selection technic employing immunomagnetic beads (Miltenyi Biotech; Bergisch Gladbach, Germany) conjugated with anti-HLA class I antibody (clone W6/32; BioLegend; San Diego, CA), as previously reported [8].

### Trophoblast culture

Purified pCTBs were plated onto uncoated multi-well plates (IWAKI; Tokyo, Japan) at a density of 1.0 x 10^6^ cells/ml (100 μl/well for 96-well plates and 500 μl/well for 24-well plates) and incubated in a humidified incubator (95% air and 5% CO2) at 37°C for 96 h in complete medium (cell-culture IMDM with 10 ng/ml epidermal growth factor (EGF; PeproTech; Rocky Hill, NJ)) with and without various concentrations of a ROCK inhibitor, Y-27632 (0.1 μM to 10 μM; 10 μM is the commonly used working concentration [21]) (Nacalai Tesque; Kyoto, Japan). EGF was added to the cell-culture IMDM with the aim of improving pCTB differentiation [10, 11]. Four hours after plating, the pCTBs were washed twice with PBS, and the medium was changed. Contaminating STB fragments were removed by this process [22]. The culture medium was changed every 48 h. To evaluate the effects of cell density, the same experiments were also performed using 0.25–4.0 x 10^6^ pCTBs/ml.

In some experiments, 50 μM of a Ras-related C3 botulinum toxin substrate 1 (Rac1) inhibitor, NSC-23766 (IC_50_: 50 μM; Merck Millipore-Calbiochem; Darmstadt, Germany), or 10 μM of a protein kinase A (PKA) inhibitor, H-89 (IC_50_: 50 nM; Biomol Research Labs, Inc.; Plymouth Meeting, PA), was also added to the pCTB culture. The optimal concentrations of these inhibitors were decided based on their IC_50_ and the findings of previous studies [23, 24].

### Measurement of adhesion cell numbers

pCTBs were cultured in uncoated 96-well plates for 24 h, washed with PBS, mounted on a coverslip with mounting reagent, and viewed with a BZ-X710 (Keyence; Osaka, Japan). Nuclei per adhered pCTB area were counted and expressed as adhesion cell numbers.

### Immunofluorescence staining for evaluation of cytoplasmic area, Ki-67, and pCTB fusion

pCTBs were cultured in uncoated 96-well tissue culture plates with and without 10 μM Y-27632 for up to 96 h. Cytoskeleton protein F-actin was stained with Alexa Fluor 488-conjugated phalloidin (Life Technologies) according to the manufacturer’s instructions. The cytoplasmic area was calculated using Hybrid Cell Count software (Keyence).

pCTBs were cultured in uncoated 96-well tissue culture plates (Iwaki) and stained with Alexa Fluor 555 Mouse anti-Ki-67 (BD Bioscience; San Jose, CA) in accordance with the manufacturer’s instructions at culture time points of 24, 48, 72 and 96 h. Cell nuclei were stained with 4',6-diamidino-2-phenylindole (DAPI; Dojindo Laboratory; Kumamoto, Japan). HeLa cells (maintained in DMEM with 10% FBS and plated at a density of 1 x 10^4^ cells/well) were used as a positive control.

pCTB fusion was evaluated as described previously [25], with slight modification. Briefly, to visualize cell boundaries, plasma membrane protein desmoplakin was stained by immunofluorescence staining. pCTBs were fixed with methanol for 30 min at room temperature, followed by blocking with 5% goat serum in PBS for 30 min at room temperature. The pCTBs were stained overnight with anti-desmoplakin antibody (0.5 μg/ml clone DP2.15; Boehringer Mannheim Biochemicals; Indianapolis, IN) at 4°C, followed by staining with Alexa Fluor 555-conjugated mouse anti-goat antibody (Invitrogen; Carlsbad, CA) as a secondary antibody. Fusion indices were calculated as the ratio of the number of fused cell nuclei divided by the number of total nuclei. A fused cell was defined as at least three nuclei surrounded by a plasma membrane.

In each staining, cell nuclei were stained with DAPI. The cells were viewed on a BZ-X710 (Keyence).

### Cell viability assay

Cell viability was determined by measuring mitochondrial activity using a WST-8 Cell Counting Kit (Nacalai Tesque) according to the manufacturer's instructions. Cell viability was expressed as the optical density (O.D.), measured with a microplate reader (FlexStation 3; Molecular Devices; CA) at 450 nm wavelength.

### Measurement of [^3^H] thymidine uptake

pCTBs were cultured in 96-well tissue culture plates. One μCi [^3^H] thymidine (Amersham; Tokyo, Japan) was added to the pCTB culture medium for 6 h at culture times of 24, 48, 72 and 96 h. HeLa cells (plated at a density of 1 x 10^4^ cells/well) were used as a positive control. Cells were harvested using UniFilter-96 (PerkinElmer Life Sciences; Waltham, MA), and [^3^H] thymidine uptake was then measured using a liquid scintillation spectrometer (Top-Count NXT; PerkinElmer Life Sciences).

### Quantitative real-time PCR

pCTBs were cultured in 24-well tissue culture plates and harvested after 96 h, and total RNA was extracted with an RNeasy Mini Kit (Qiagen; Valencia, CA) and digested with RNase-free DNase I (Qiagen) in accordance with the manufacturer’s instructions. First-strand cDNA was synthesized using an iScript cDNA Synthesis Kit (Bio-Rad). Quantitative real-time PCR (qPCR) was performed with Thunderbird SYBR qPCR Mix (Toyobo; Tokyo, Japan) and a CFX-96 real-time PCR system (Bio-Rad). The relative gene expression was normalized against expression of a housekeeping gene, ribosomal phosphoprotein, large, P0 (RPLP0). PCR primers were designed as shown in Table 1 and synthesized at Fasmac (Kanagawa, Japan). All the primer sequences were validated using the Basic Local Alignment Search Tool (National Center for Biotechnology Information) in order to ensure their specificity, and by melting curve analysis to confirm the amplification of single targets.

**Table 1:** Primer sequences.

NM number	Gene Name	Forward primer	Reverse primer
NM_053275	RPLP0	TTCGACAATGGCAGCATCTACAA	CTGCAGACAGACACTGGCAACA
NM_001130925.1	Syncytin1	ATTTTGGCAACCACGAACGG	AGCCACTTTAACCGCAGTTG
NM_207582.2	Syncytin2	TCAAATGGTGCAGTGACTCG	AAGGGCTTTTCCACAGCTTC
NM_000737.3	CGB	GTGTGCATCACCGTCAACAC	TAGTTGCACACCACCTGAGG
NM_003643.3	GCM1	TGAAACTGCCACAGAACGTG	TTGTTGGTATTGCGCATGGC
NM_002127.5	HLA-G	TTGCAGCTGTAGTCACTGGAG	AGGGCAGCTGTTTCACATTG

RPLP0, ribosomal phosphoprotein, large, P0; CGB, chorionic gonadotropin beta chain; GCM1, glial cell missing 1; HLA-G, human leukocyte antigen-G.

### Measurement of hCG-β secretion

After 96 h of culture (selected based on a previous report [7]), the culture supernatants were harvested, and the concentrations of hCG-β in them were measured by ELISA (DRG Diagnostics; Marburg, Germany). At the same time, whole-cell lysates were prepared with 0.4% NP40 (Particle Data Laboratories; Elmhurst, IL) in PBS, and the protein concentrations in them were measured by BCA protein assay (Pierce; Rockford, IL).

hCG-β secretion was normalized against the protein concentration in each whole-cell lysate (expressed as mIU/ml/mg protein), as described previously [26, 27].

### Statistical analysis

For analysis of images, 10 fields of view per condition were randomly selected and evaluated by blinded investigators. Student’s t-test, one-way analysis of variance (ANOVA) and Wilcoxon’s matched-pairs signed rank test were used to identify significant differences within the groups, followed by Holm-Sidak’s multiple-comparison test to determine differences between groups. Spearman’s rank correlation was applied to determine correlations between the concentrations of Y-27632 and the viability of pCTBs. All statistical analyses were performed using GraphPad Prism6 software (GraphPad Software; La Jolla, CA). Results were considered statistically significant if *P* < 0.05. All data are shown as the mean ± SD unless otherwise noted.

## Results

### Y-27632 enhanced pCTB adhesion to tissue culture plates

**Fig 1:**
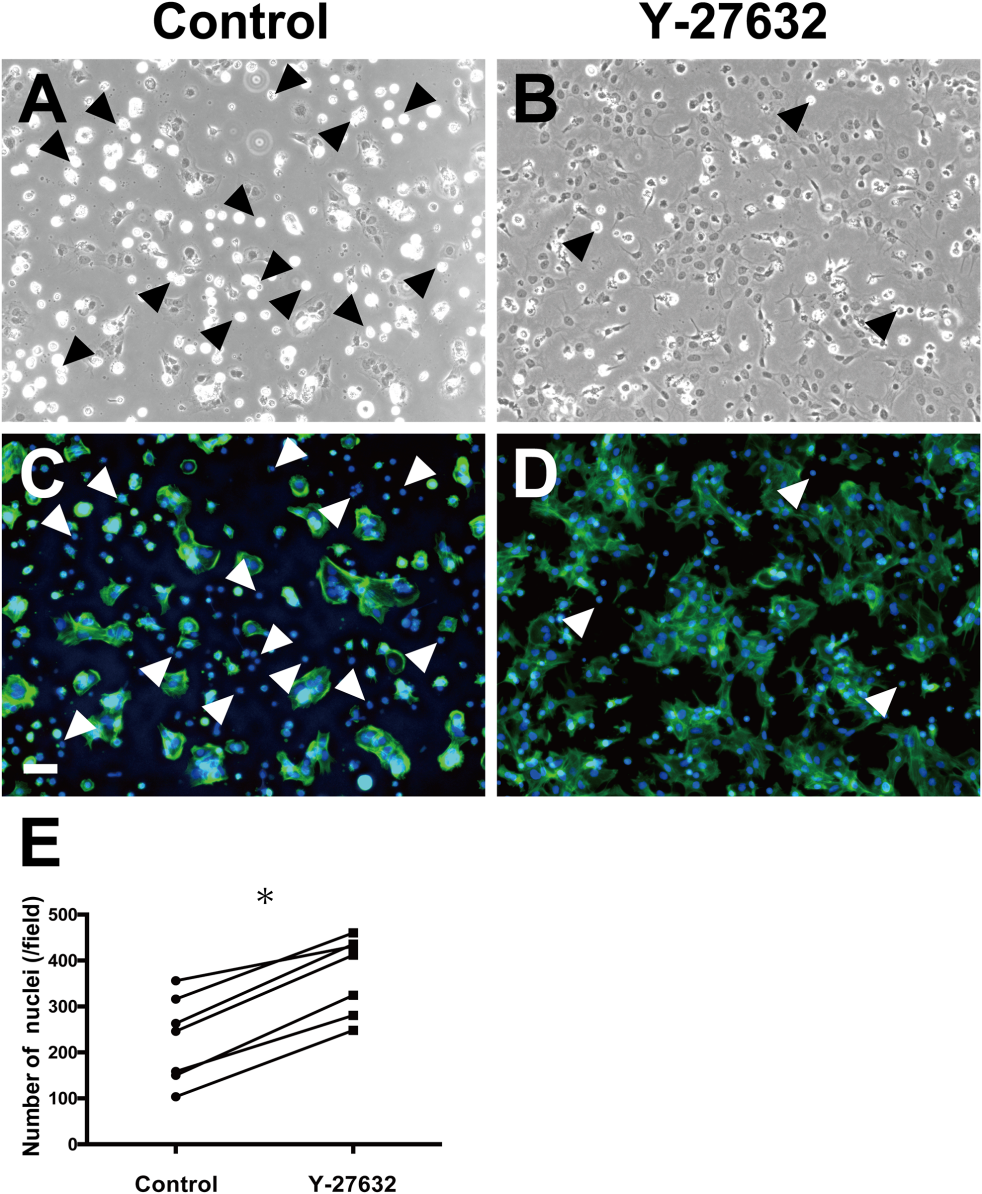
Effects of Y-27632 on pCTB adhesion. Phase-contrast (A, B) and immunofluorescence (C, D) images of pCTBs cultured for 24 h in the absence (A, C) or presence (B, D) of 10 μM Y-27632. Cytoplasmic F-actin was stained with Alexa Fluor 488-conjugated phalloidin, and nuclei were stained with DAPI. (A) and (C), and (B) and (D) show the same fields, respectively. Scale bar = 50 μm. (E) The number of adhered cell nuclei were counted per image (from separate placentas, n = 7). Each point shows the mean of 10 fields from one condition. Statistical differences were determined with Wilcoxon’s matched-pairs signed rank test. Arrowheads indicate apoptotic cells, which were excluded from the cell counts. *, *P* < 0.05.

The effect of Y-27632 on pCTB adhesion was evaluated by phase-contrast and immunofluorescence microscopy. Phase-contrast images showed that adhered pCTBs, identified by their slightly dark-gray nuclei and spreading cytoplasm, were increased in plates containing Y-27632 (Fig 1B and S1B, S1D and S1F Fig) compared with those without Y-27632 (Fig 1A and S1A, S1C and S1E Fig). The nuclei per adhered pCTB area were counted, and the data showed that Y-27632 increased pCTB adhesion to tissue culture plates (Fig 1E).

Shrunken cells with bright borders (Fig 1A and 1B, arrowheads) were annexin V-positive, suggesting that they had undergone apoptosis (S2 Fig). Thus, they were excluded from further counts.

### Y-27632 enhanced pCTB viability

**Fig 2:**
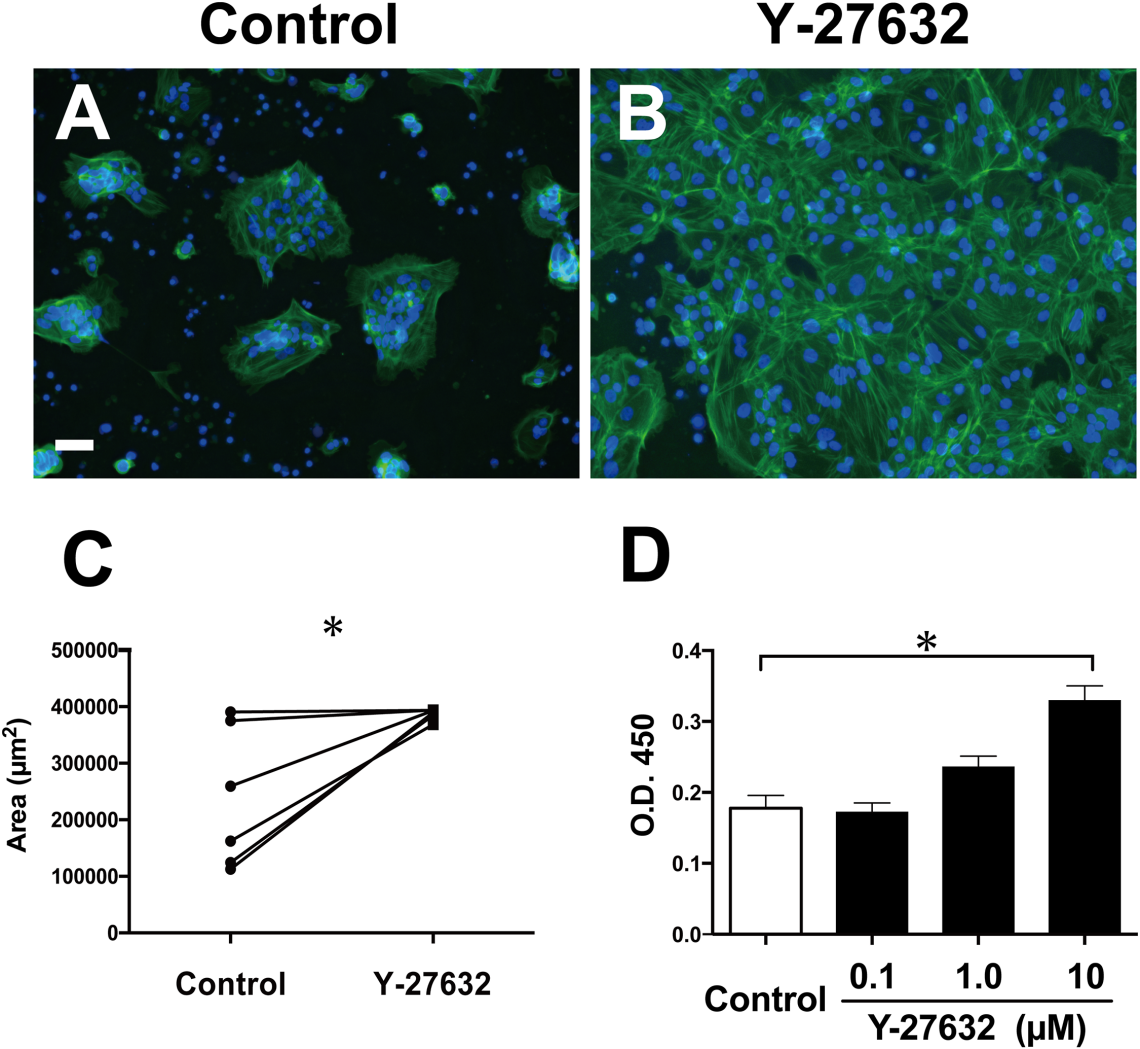
Effects of Y-27632 on pCTB viability. pCTBs were cultured in the absence (A) and presence (B) of 10 μM Y-27632 for 96 h. Cytoplasmic F-actin was stained with Alexa Fluor 488-conjugated phalloidin, and nuclei were stained with DAPI. Scale bar = 50 μm. (C) The cytoplasmic area was measured using Hybrid Cell Count software (Keyence). Data are expressed as μm^2^. Each point shows the mean of 10 fields from one condition (from six separate placentas, n = 6). Statistical differences were determined with Wilcoxon’s matched-pairs signed rank test. (D) Mitochondrial activity was measured by WST-8 assay, and data are expressed as O.D. 450 nm. The data are from 3 separate experiments using pCTBs from 3 different placentas. All data are shown as the mean ± SD. *, *P* < 0.05.

To evaluate the effect of Y-27632 on pCTB viability, we compared the cytoplasmic area and mitochondrial activity of pCTBs cultured with and without Y-27632 for 96 h. There were morphological differences between pCTBs cultured with and without Y-27632. pCTBs cultured without Y-27632 were seen to be aggregated and shrunken (Fig 2A), whereas pCTBs cultured with Y-27632 formed a cytoplasmic monolayer and looked nearly confluent (Fig 2B). These differences were evaluated by calculating the cytoplasmic area, and the data showed that Y-27632 significantly increased the cytoplasmic area of pCTBs (*P* < 0.05) (Fig 2C). The cytoplasmic area of pCTBs cultured with Y-27632 plateaued because all of the fields were nearly confluent. Y-27632 also significantly enhanced pCTB mitochondrial activity in a dose-dependent manner (*P* < 0.05) (Fig 2D).

### Y-27632 did not affect pCTB proliferation

**Fig 3:**
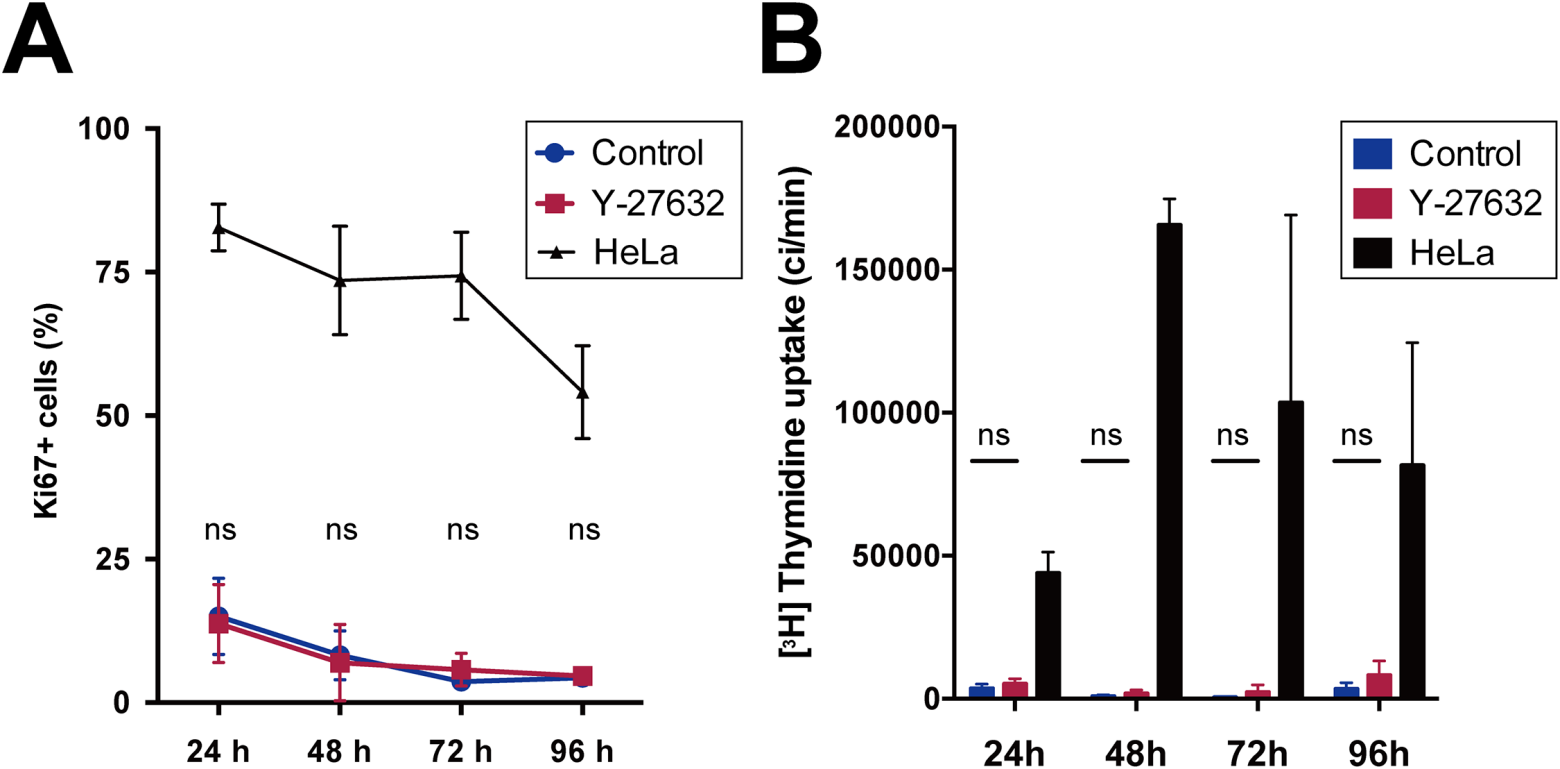
The effect of Y-27632 on pCTB proliferation. pCTBs were cultured with and without Y-27632 (10 μM) for 24, 48, 72 and 96 h. HeLa cells were also cultured and tested as a positive control. (A) Ki-67 protein in pCTBs and HeLa cells was detected by immunofluorescence staining. The Ki-67-positive cell number was divided by the total nuclei number and shown as Ki-67+ cells. Each point was the average of pCTBs from 3 different donors, and one condition was the mean of 10 randomly selected fields in one well. The HeLa cell data were the mean of 10 randomly selected fields. (B) [^3^H] thymidine was added to the culture medium of pCTBs with and without Y-27632 and HeLa cells for 6 hours. Each dot in the figure represents the mean of pCTBs from 4 different donors, and the data of each donor were the mean of triplicate determinations. The HeLa cell data are the mean of triplicate determinations. Data are expressed as the mean ± SD. Statistical analyses were performed only between pCTBs with and without Y-27632 by using Student’s t-test with correction for multiple comparisons using Holm-Sidak method. ns, not significant.

Y-27632 reportedly improved hCEC proliferation [28]. Basically, the adhered cell number and cell viability measurements are affected by the cells’ potential to proliferate. Therefore, we evaluated the effect of Y-27632 on pCTB proliferation by immunofluorescence staining for Ki-67 and [^3^H] Thymidine uptake at various culture time-points. There were no significant differences in the Ki-67–positive cell ratio (Fig 3A) or [^3^H] thymidine uptake (Fig 3B) between pCTBs cultured with and without Y-27632. In addition, as shown in Fig 3A, only 15% of pCTBs were Ki-67–positive at 24 h, and the ratio of Ki-67–positive pCTBs gradually decreased with time. The absolute values of the Ki-67+ cell ratio as well as [^3^H] thymidine uptake by pCTBs were very low compared with rapidly proliferating HeLa cells (Fig 3A and 3B). These results suggest that Y-27632 did not affect pCTB proliferation, and that pCTB proliferation was limited, as previously reported [11].

### Y-27632 enhanced pCTB differentiation

CTB differentiation includes two different steps: morphological differentiation (syncytialization) and biochemical differentiation (production of hCG and other placenta-specific hormones) [29]. We separately examined whether Y-27632 enhances both of those steps.

**Fig 4:**
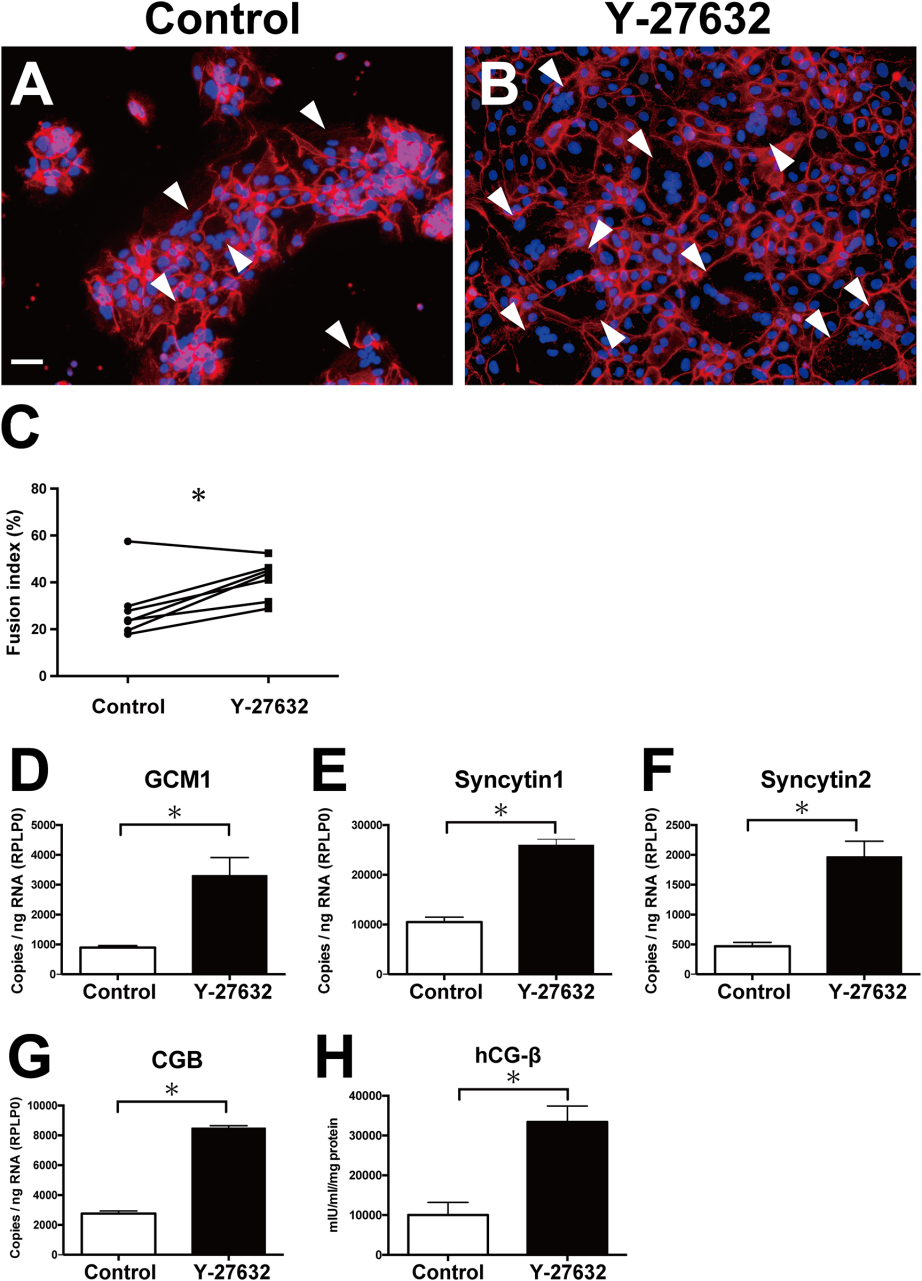
Effects of Y-27632 on pCTB differentiation. (A, B) pCTBs were cultured in the absence (A) and presence (B) of 10 μM Y-27632 for 96 h. Plasma membrane protein desmoplakin I was stained with Alexa Fluor 555, and nuclei were stained with DAPI. Arrowheads indicate fused, large, cytoplasmic multinucleated syncytiotrophoblast-like cells. Scale bar = 50 μm. (C) The fusion index was calculated using pCTBs from 6 different donors. Each point shows the mean of 10 fields in one well. (D-G) Total RNA was extracted from pCTBs cultured in the presence and absence of 10 μM Y-27632 for 96 h, and qPCR was used to quantify mRNA for GCM1 (D), syncytin-1 (E), syncytin-2 (F) and CGB (G). The experiments were performed in triplicate, and the data are representative of 3 separate experiments using pCTBs from 3 different donors. (H) pCTBs were cultured in the presence and absence of 10 μM Y-27632 for 96 h. Protein levels of hCG-β in the culture supernatant were measured by ELISA. The concentration of hCG-β was normalized against the protein concentration in the whole cell lysate (as mIU/ml/mg protein). The experiments were performed in triplicate, and the data are representative of 3 separate experiments using pCTBs from 3 different donors. Data are expressed as the mean ± SD. *, *P* < 0.05.

Immunofluorescence staining was performed to evaluate the morphological differentiation, i.e., syncytialization, of pCTBs when cultured with and without Y-27632 for 96 h. Y-27632 enhanced formation of multinucleated, wide-spread syncytia (Fig 4B) compared with in its absence (Fig 4A). The fusion index of pCTBs cultured with Y-27632 was significantly higher than in its absence (*P* < 0.05) (Fig 4C).

To confirm syncytialization at the mRNA level, mRNA expression of glial cell missing (GCM)1, syncytin-1 and syncytin-2 was examined by qPCR [30–32]. Y-27632 significantly enhanced the mRNA expression levels of GCM1, syncytin-1 and syncytin-2 (*P* < 0.05; Fig 4D–4F).

qPCR and ELISA were used to investigate whether Y-27632 facilitates biological differentiation of pCTBs by measuring, respectively, the mRNA expression of chorionic gonadotropin beta chain (CGB) and the hCG-β protein concentration in the culture supernatant at 96 h. Y-27632 significantly enhanced both of those indicators of biochemical differentiation (*P* < 0.05; Fig 4G and 4H).

### Impact of cell density on the effects of Y-27632

**Fig 5:**
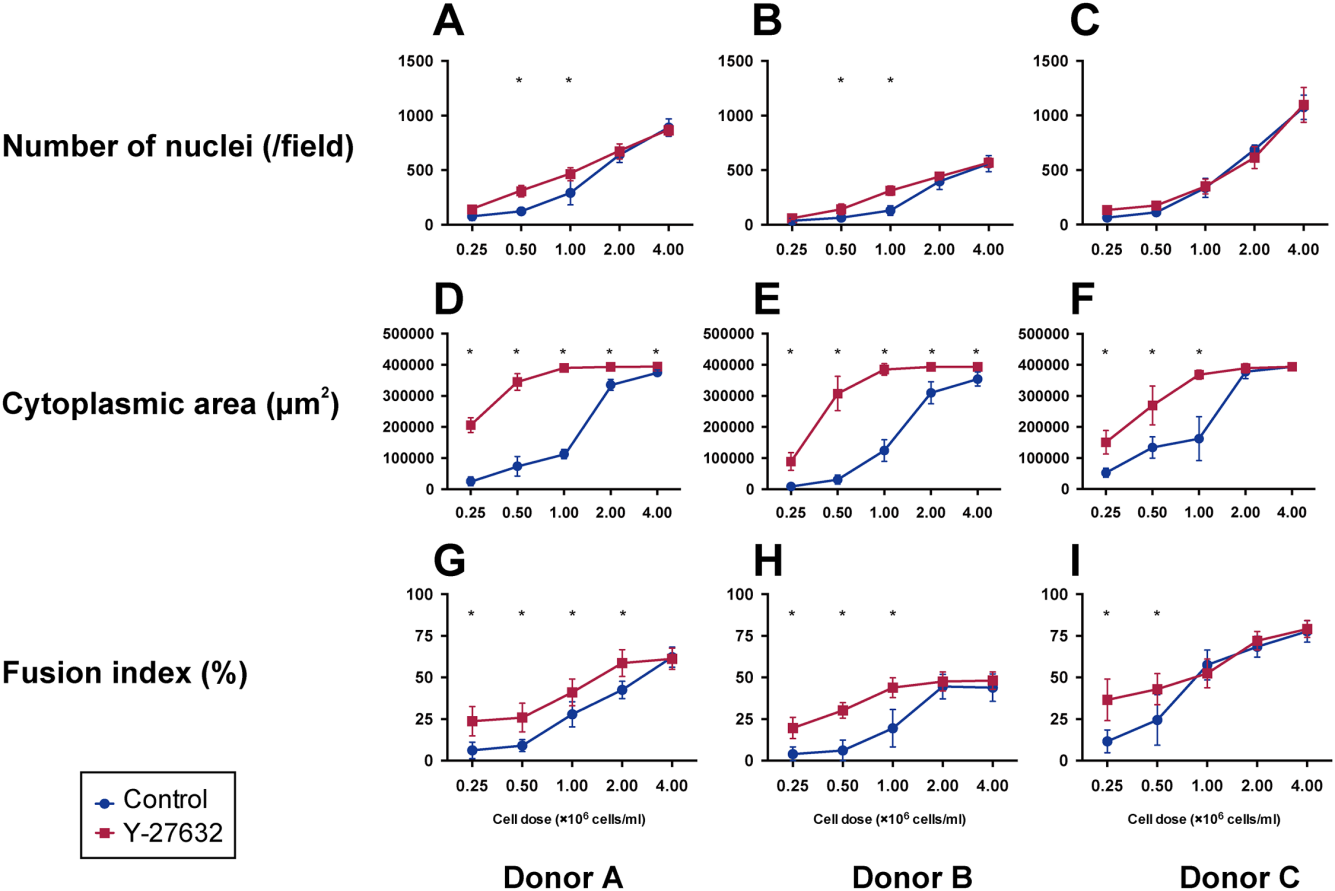
Effects of cell density and Y-27632 on pCTB culture. pCTBs (from 3 different donors) were cultured with and without Y-27632 (10 μM) at cell densities of 0.25–4.0 x 10^6^ cells/ml for 96 h. Each cell was subjected to immunofluorescence staining, and the number of nuclei (A-C), cytoplasmic area (D-F) and fusion index (G-I) were determined. Each dot shows the mean of 10 fields of view in each well. Data are expressed as the mean ± SD. Statistical analyses were performed between with and without Y-27632 by using Student’s t test with correction for multiple comparisons using the Holm-Sidak method. *, significantly different (*P* < 0.05).

There are two possibilities for how Y-27632 improved pCTB viability and fusion: One is a direct effect of Y-27632 itself, and the other is an indirect effect arising from the increased number of adhered pCTBs. To clarify the impact of the cell density on the effects of Y-27632 in pCTB culture, pCTBs were seeded at densities of 0.25–4.0 x 10^6^ cells/ml with and without Y-27632 (10 μM) for 96 h. Then the number of nuclei and cytoplasmic area were determined, and the fusion index was calculated (n = 3) (Fig 5).

The numbers of nuclei of pCTBs cultured with and without Y-27632 increased in a density-dependent manner, and they were significantly increased at densities of 0.5 x 10^6^ cells/ml and 1.0 x 10^6^ cells/ml in donors A and B (Fig 5A and 5B). There were no significant differences between with and without Y-27632 at the lowest (0.25 x 10^6^ cells/ml) or highest (2.0 and 4.0 x 10^6^ cells/ml) densities in any of the donors (Fig 5A–5C).

The cytoplasmic area also increased in a density-dependent manner, and Y-27632 significantly increased the area at densities of 0.25, 0.5 and 1.0 x 10^6^ cells/ml in all donors (Fig 5D–5F). pCTBs cultured with Y-27632 at densities of 1.0–4.0 x 10^6^ cells/ml looked nearly confluent.

The fusion index also tended to increase in a density-dependent manner, and Y-27632 significantly increased the index at densities of 0.25–2.0 x 10^6^ cells/ml in donor A (Fig 5G), 0.25–1.0 x 10^6^ cells/ml in donor B (Fig 5H) and 0.25–0.5 x 10^6^ cells/ml in donor C (Fig 5I). These results suggest that although the baseline cytoplasmic area and fusion index vary among donors, Y-27632 was effective, especially at lower cell densities, but not at higher cell densities.

### Effects of Rac1 and PKA inhibitors on Y-27632-induced pCTB differentiation

**Fig 6:**
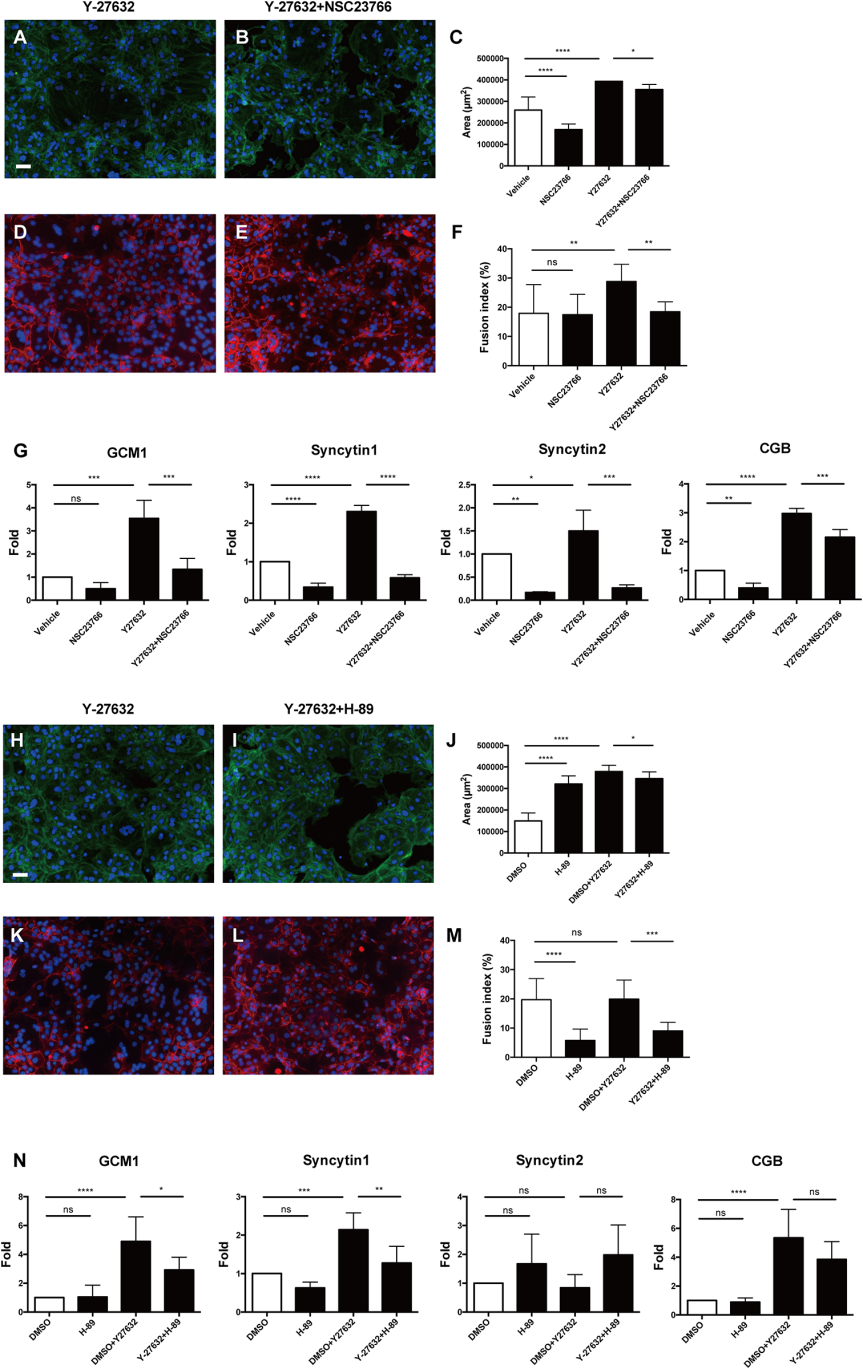
Effects of a Rac 1 inhibitor, NSC-23766, and a PKA inhibitor, H-89, on fusion and expression of fusogenic genes and hCG-β in pCTBs. **(A-G)** pCTBs were cultured in the presence and absence of 10 μM Y-27632 and/or 50 μM NSC-23766 for 96 h. (A, B) Cytoplasmic F-actin was stained with Alexa Fluor 488-conjugated phalloidin, and nuclei were stained with DAPI. (A) pCTBs cultured with Y-27632 (10 μM), (B) pCTBs cultured with Y-27632 (10 μM) and NSC-23766 (50 μM). Scale bar = 50 μm. (C) The cytoplasmic area was measured using Hybrid Cell Count software (Keyence). Each bar shows the mean of 10 fields in each well, and the data are representative of the experiment using 3 pCTBs from 3 different donors. (D, E) Plasma membrane protein desmoplakin I was stained with Alexa Fluor 555, and nuclei were stained with DAPI. (D) pCTBs cultured with Y-27632 (10 μM), (E) pCTBs cultured with Y-27632 (10 μM) and NSC-23766 (50 μM). Scale bar = 50 μm. (F) The fusion index was calculated. Each column shows the mean of 10 fields in one well, and the data are representative of the experiment using 3 pCTBs from 3 different donors. (G) Total RNA was extracted, and the mRNA expression levels for GCM1, syncytin-1, syncytin-2 and CGB are shown. Each column shows the mean of the results for 3 pCTBs from 3 different donors, and the fold change was calculated against each control sample. (H-N) pCTBs were cultured in the presence and absence of 10 μM Y-27632 and/or 10 μM H-89 for 96 h. (H, I) Cytoplasmic F-actin was stained with Alexa Fluor 488-conjugated phalloidin, and nuclei were stained with DAPI. (H) pCTBs cultured with Y-27632 (10 μM), (I) pCTBs cultured with Y-27632 (10 μM) and H-89 (10 μM). Scale bar = 50 μm. (J) The cytoplasmic area was measured using Hybrid Cell Count software (Keyence). Each column shows the mean of 10 fields in one well, and the data are representative of the experiment using 3 pCTBs from 3 different donors. (K, L) Plasma membrane protein desmoplakin I was stained with Alexa Fluor 555, and nuclei were stained with DAPI. (K) pCTBs cultured with Y-27632 (10 μM), (L) pCTBs cultured with Y-27632 (10 μM) and H-89 (10 μM). Scale bar = 50 μm. (M) The fusion index was calculated. Each column shows the mean of 10 fields in one well, and the data are representative of the experiment using 3 pCTBs from 3 different donors. (N) Total RNA was extracted, and mRNA expression levels for GCM1, syncytin-1, syncytin-2 and CGB are shown. Each column shows the mean of the results for 5 pCTBs from 5 different donors, and the fold change was calculated against each control sample. Error bar shows SD. *, *P* < 0.05. **, *P* < 0.005. ***, *P* < 0.0005. ****, *P* < 0.0001. ns, not significant.

We next investigated the pathway of Y-27632’s effects on pCTB differentiation. Previous studies suggested that Y-27632 inhibits the Rho/ROCK pathway and induces activation of Rac1 (one of the effector proteins of the Rho family) in hESC, since there is antagonism between Rac1 and the Rho/ROCK pathway [12, 13, 33]. Therefore, we examined whether Y-27632’s effects on pCTB differentiation occurred via Rac1 activation. NSC-23766 (50 μM), an inhibitor of Rac1, was used in these experiments. The effect of 50 μM NCS-23766 on the cytoplasmic area of pCTBs cultured with Y-27632 was small, but statistically significant (Fig 6A–6C).

NSC-23766 significantly diminished Y-27632-induced pCTB fusion (*P* < 0.05; Fig 6D–6F) and mRNA expression for each of GCM1, syncytin-1, syncytin-2 and CGB (*P* < 0.05; Fig 6G). It also abrogated spontaneous expression of syncytin-1, syncytin-2 and CGB (*P* < 0.05).

H-89, an inhibitor of PKA, was used to confirm whether the effects of Y-27632 are exerted via the conventional CTB differentiation pathway, which is a downstream signaling pathway of PKA [34]. The effect of 10 μM of H-89 on the cytoplasmic area of pCTBs cultured with Y-27632 was small but statistically significant (Fig 6H–6J). We found that 10 μM H-89 significantly diminished Y-27632-induced fusion (*P* < 0.05; Fig 6K–6M) and mRNA expression for GCM1 and syncytin-1 (*P* < 0.05; Fig 6N).

mRNA expression of a housekeeping gene (RPLP0) was not affected by NSC-23766 or H-89, suggesting that they do not suppress general transcription of pCTBs (S3A and S3B Fig). Expression of HLA-G, a marker gene of extravillous cytotrophoblasts [35], was not affected by these inhibitors. Moreover, HLA-G copy numbers were very low, suggesting that pCTBs treated with those inhibitors remained in the CTB phenotype and did not differentiate into extravillous trophoblasts (S3C and S3D Fig). The Ki-67–positive cell ratio was not affected by either inhibitor, suggesting that they did not induce pCTB proliferation (S3E and S3F Fig).

## Discussion

This study aimed to enhance pCTB adhesion to tissue culture plates and their differentiation. We found that Y-27632 significantly enhanced adhesion, viability, syncytialization and hCG-β production of pCTBs.

In agreement with current knowledge, the extent of spontaneous adhesion of pCTBs to uncoated plates varied among individuals (S1 Fig). We found that Y-27632 significantly enhanced pCTB adhesion to uncoated tissue culture plates (Fig 1), as it did for hESC and hCEC. In those cells, Y-27632 inhibited ROCK activation and induced Rac 1 activation, thereby preventing apoptosis and improving their culture efficiency [12, 15]. We observed round, shrunken cells with very bright borders (Fig 1A and 1B, arrowheads) in pCTBs cultured with and without Y-27632. Those cells were annexin V-positive (S2 Fig), lacked cytoplasm and later detached from the culture plates, suggesting that they were apoptotic cells. Therefore, we hypothesized that Y-27632 reduced apoptosis and increased adhesion of pCTBs by the same mechanisms as in hCEC and hESC cultures. However, we were unable to detect any significant differences in the number of those cells between the presence and absence of Y-27632. Future studies are needed to clarify the mechanism of Y-27632’s effect on pCTB adhesion. Y-27632 also enhanced pCTB viability, assessed by the cytoplasmic area and mitochondrial activity (Fig 2).

It was reported that 85% of purified CTBs were in G0/G1 phase [7] and underwent a rapid decline in expression of proliferation markers [11]. We found that proliferation of the pCTBs used in our experiment was also limited, and Y-27632 did not affect proliferation during at least the first 96 h of culture. This suggests that Y-27632’s effects on pCTB adhesion and viability were derived purely from the direct action of Y-27632.

**Fig 7:**
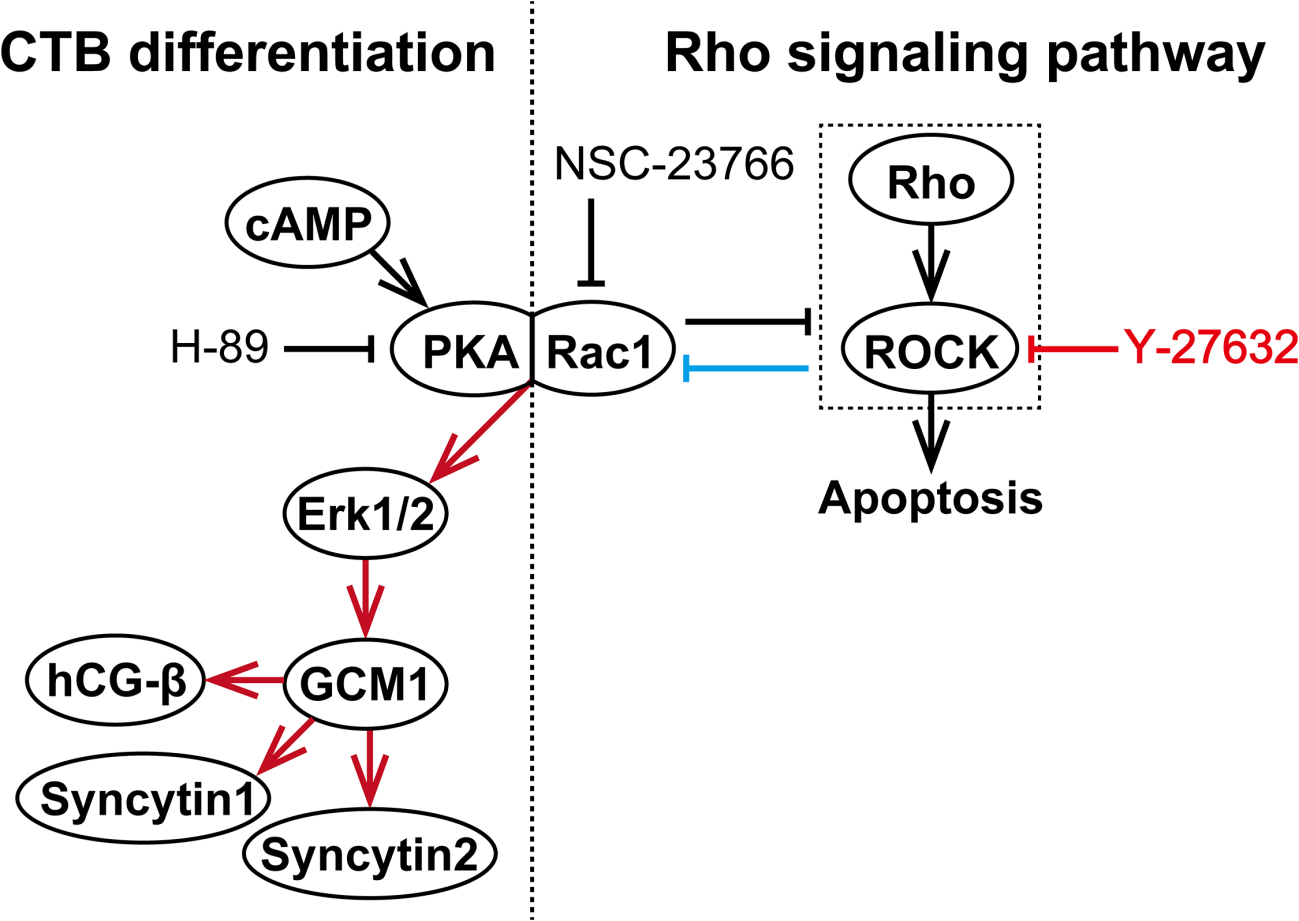
The putative signaling system of Rho signaling pathway and CTB differentiation pathway. (Right side) Rho signaling pathway. There is antagonism between Rac1 and the Rho/ROCK pathway, and Y-27632 increases Rac1 activity by inhibiting ROCK. (Left side) CTB differentiation pathway. cAMP activates PKA. PKA then activates GCM1 via Erk 1/2, thereby inducing CTB differentiation. Rac1 and PKA interact directly and regulate the downstream signal cascade, especially to Erk 1/2.

Previous studies provided evidence regarding the signal transduction pathways for differentiation of CTBs to STBs (Fig 7). Briefly, intracellular cAMP-elevating stimulation such as G-protein-coupled receptor stimulation by hCG-β activates PKA [34, 36–38]. PKA activates GCM1 via Erk 1/2. GCM1 then acts as the transcription factor for two endogenous retrovirus elements, syncytin-1 and syncytin-2, which finally promote CTB differentiation [30–32, 39]. To test whether Y-27632 promotes pCTB differentiation, we investigated that differentiation from its morphological and biological aspects. We found that Y-27632 significantly promoted differentiation of pCTBs, which was confirmed by increased syncytialization, expression of fusogenic genes and hCG-β production (Fig 4). These results indicated that Y-27632 can activate the GCM1-syncytin pathway, similar to the molecular mechanism previously reported in STBs [31, 32].

Previous studies evaluated pCTB fusion at time points of 66 h [27], 72 h [40], 96 h [41] and 120 h [42]. Meanwhile, hCG-β secretion was evaluated at time points from 66 h to 96 h [7, 10]. In addition, we found that hCG-β secretion from pCTBs was increased until 144 h regardless of addition of Y-27632, and the effect of Y-23632 was obvious at 96 h (S4 Fig). This suggests that the fusion and hCG-β production processes of pCTBs and their viability are maintained at least until 96 h. For these reasons, we selected 96 h for evaluating pCTB differentiation, as in previous studies [7, 10, 41].

The fusion index is usually used to evaluate pCTB fusion [25, 27, 43]. The fusion index values reported in this study varied among the donors and were a little lower than reported in other studies (Fig 4). Higher cell densities raised the fusion index values to the vicinity of those reported earlier (Fig 5). Therefore, differences in cell density may be one factor underlying this discrepancy. In addition, the method for fusion index calculation, the evaluation time point and the pCTB culture conditions varied among the studies. Those differences also may be potential biases for the fusion index.

To clarify whether the effect of Y-27632 was a direct effect or not, we observed the impact of the cell density on Y-27632’s effects (Fig 5). The adhered cell number, cytoplasmic area and fusion index each increased in a cell density-dependent manner with and without Y-27632 (Fig 5), suggesting that increasing the viable cell density is one important factor for improving pCTB viability and differentiation. Moreover, although there was much donor variability, Y-27632 tended to have greater effects on the cytoplasmic area and fusion index at lower cell densities. Interestingly, although the number of nuclei was not significantly increased by Y-27632 at a density of 0.25 x 10^6^ cells/ml, the cytoplasmic area and fusion index were significantly increased in all 3 donors. Those results suggest that Y-27632 not only increases viable adhered cells but also induces differentiation itself. On the other hand, the effects of Y-27632 disappeared at higher cell densities. In dissociation cultured hESC, abnormal ROCK activation and apoptosis were induced by loss of Ca^2+^-dependent intercellular adhesion, and Y-27632 inhibited this apoptosis signal [12]. This may partially explain why the effects of Y-27632 disappeared at higher cell densities. A high cell density may enable pCTBs to contact each other easily and promote cell–cell adhesion, leading to pCTB survival and fusion even without Y-27632. E-cadherin, one of the key molecules in cell–cell adhesion, was remodeled and decreased during the process of trophoblast fusion [44, 45]. However, the roles of E-cadherin in trophoblast survival and initiation of fusion are not well understood. This is another interesting point worthy of investigation in future studies.

A number of studies have shown that intracellular cAMP-elevating reagents such as forskolin and cAMP itself can trigger primary human CTB and trophoblast cell line BeWo fusion, mainly via PKA signaling. Although our preliminary experiments confirmed that addition of a cAMP analog, 8-Br-cAMP, promoted pCTB fusion and maturation, as previously reported [46], it reduced pCTB viability (S5 Fig). At high concentration, forskolin reportedly shows some cytotoxicity to some cells [47]. Also, 8-Br-cAMP induces CTB apoptosis together with CTB differentiation in serum-containing medium [36], making it unsuitable for use in long-term culture. In contrast, our WST-8 assay (Fig 2) confirmed that Y-27632 causes no remarkable impairment of cell viability.

In order to clarify the signal transduction pathway employed by Y-27632, we used two specific inhibitors: NSC-23766, an inhibitor of Rac 1, and H-89, an inhibitor of PKA. As expected, NSC-23766 significantly diminished Y-27632-induced pCTB fusion and expression of fusogenic genes and CGB (Fig 6). These results suggest that Y-27632 promotes pCTB differentiation via Rac 1.

Interestingly, NSC-23766 abrogated expression of fusogenic genes and CGB in the absence of Y-27632 (Fig 6), suggesting that Rac1 plays a role in spontaneous differentiation of pCTBs. It also strongly suggests that the balance in Rac1/ROCK activity may regulate differentiation of CTBs, presumably because Rac1 and ROCK regulate each other [13]. As a matter of fact, a Rho family molecule, RhoE, participated in fusion of a trophoblast cell line, BeWo [48]. However, ours is the first report suggesting Rac1/ROCK involvement in primary CTB fusion. Our results are in agreement with an earlier clinical finding of increased ROCK 2 expression in preeclamptic placentas [49]. This notion is also supported by findings that Rac1 mediated membrane-type matrix metalloproteinase 1 (MT1-MMP)-induced myeloid cell fusion [50] and that MT1-MMP contributed to trophoblast fusion [51]. The mechanisms controlling spontaneous Rac1/ROCK activation in CTBs and their role in placental biology/pathology warrant further study.

Finally, H-89 significantly diminished Y-27632-induced GCM1 and Syncytin-1 activation as well as pCTB fusion (Fig 6), suggesting that Y-27632 activates physiological PKA signals [34, 36–38], at least in pCTB fusion. This fact is consistent with a previous report that Rac1 and PKA interact directly to form a multifunctional scaffold and regulate the downstream signal cascade, especially to Erk 1/2 [52]. Although it was not statistically significant, H-89 marginally diminished Y-27632-induced CGB mRNA expression.

Some limitations of this study are as follows. Because Y-27632 is a synthetic signaling modifier, it may lead to off-target effects in some experiments. For example, if Y-27632 were present when analyzing pCTBs derived from a diseased placenta with an impaired Rho signaling pathway, the existence of that impairment in the pCTBs might be masked. In addition, the applicability of pCTBs derived from diseased placentas should be tested. In this context, it will be interesting to examine if administration of Y-27632 can improve the dysfunction of pathological placentas.

## Conclusions

Y-27632 enhanced pCTB adhesion, viability and differentiation via the conventional CTB differentiation pathway. This suggests that addition of Y-27632 to cultures may be an effective method for creating a stable culture model for studying the biology of CTBs and STBs *in vitro*.

## Supporting information

S1 Fig Individual variation in pCTB adhesion ability. Three different placenta-derived pCTBs were cultured with (B, D and F) and without (A, C and E) Y-27632 for 24 h. They were washed with PBS, mounted on a coverslip with mounting reagent and viewed by phase-contrast microscopy. A and B, C and D, and E and F were derived from the same placentas, respectively.
(TIF)

S2 Fig Detection of annexin V-positive apoptotic cells. pCTBs were cultured with (B) and without (A) 10 μM Y-27632 for 12 h. Exposed phosphatidylserine, which occurs early in apoptosis, was detected with an FITC-conjugated annexin V (Annexin V assay kit (MBL; Nagoya, Japan)) in accordance with the manufacturer’s instructions.
(TIF)

S3 Fig Effects of NSC-23766 and H-89 on mRNA expression for RPLP0 and HLA-G, and Ki-67 expression. pCTBs were cultured with and without Y-27632, NSC-23766 (A, C, E) and H-89 (B, D, F) for 96 h. (A-D) Total RNA was extracted, and the mRNA expression levels for RPLP0 and HLA-G are shown. (E, F) Ki-67 of pCTBs was detected by immunofluorescence staining. The Ki-67–positive cell number was divided by the total nuclei number and shown as Ki-67+ cells. Each bar shows the mean of the results for 3 pCTBs from 3 different donors. Data are expressed as the mean ± SD. ns, not significant.
(TIF)

S4 Fig Time-course analysis of hCG-β production by pCTBs. pCTBs were cultured with and without Y-27632 for 144 h. Culture supernatants were collected every 48 h, and the concentration of hCG-β was measured by ELISA. Each column shows the mean of the results for 3 pCTBs from 3 different donors. Data are expressed as the mean ± SD.
(TIF)

S5 Fig The effect of cAMP in pCTB viability. pCTBs were cultured with and without 8-Br-cAMP (10 μM) for 96 h. Cell viability was evaluated by WST-8 assay. The experiment was performed in quintuplicate. Data are expressed as the mean ± SD. *, *P* < 0.05.
(TIF)

S1 Table Individual data depicted in Fig 1. (XLSX)

S2 Table Individual data depicted in Fig 2. (XLSX)

S3 Table Individual data depicted in Fig 3. (XLSX)

S4 Table Individual data depicted in Fig 4. (XLSX)

S5 Table Individual data depicted in Fig 5. (XLSX)

S6 Table Individual data depicted in Fig 6. (XLSX)

S7 Table Individual data depicted in S3 Fig. (XLSX)

S8 Table Individual data depicted in S4 Fig. (XLSX)

S9 Table Individual data depicted in S5 Fig. (XLSX)
