# Supplementary figures and images for: A Rho-associated coiled-coil containing kinases (ROCK) inhibitor, Y-27632, enhances adhesion, viability and differentiation of human term placenta-derived trophoblasts in vitro
Source: PLoS One. 2017 May 19;12(5):e0177994. doi: 10.1371/journal.pone.0177994 (PMC5438149; doi:10.1371/journal.pone.0177994)

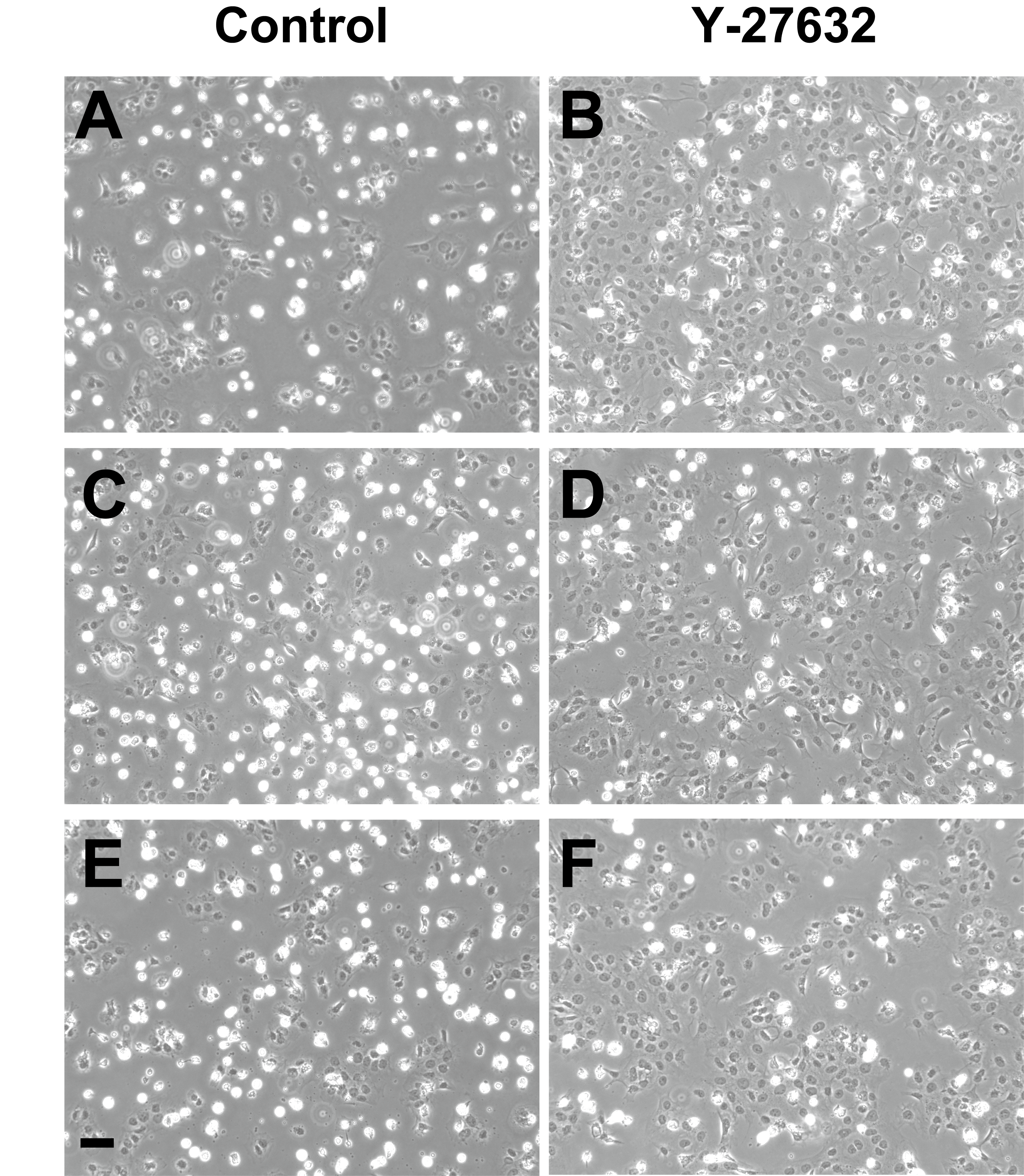

Supplement: S1 Fig — Three different placenta-derived pCTBs were cultured with (B, D and F) and without (A, C and E) Y-27632 for 24 h. They were washed with PBS, mounted on a coverslip with mounting reagent and viewed by phase-contrast microscopy. A and B, C and D, and E and F were derived from the same placentas, respectively. (TIF) [file pone.0177994.s001.tif]

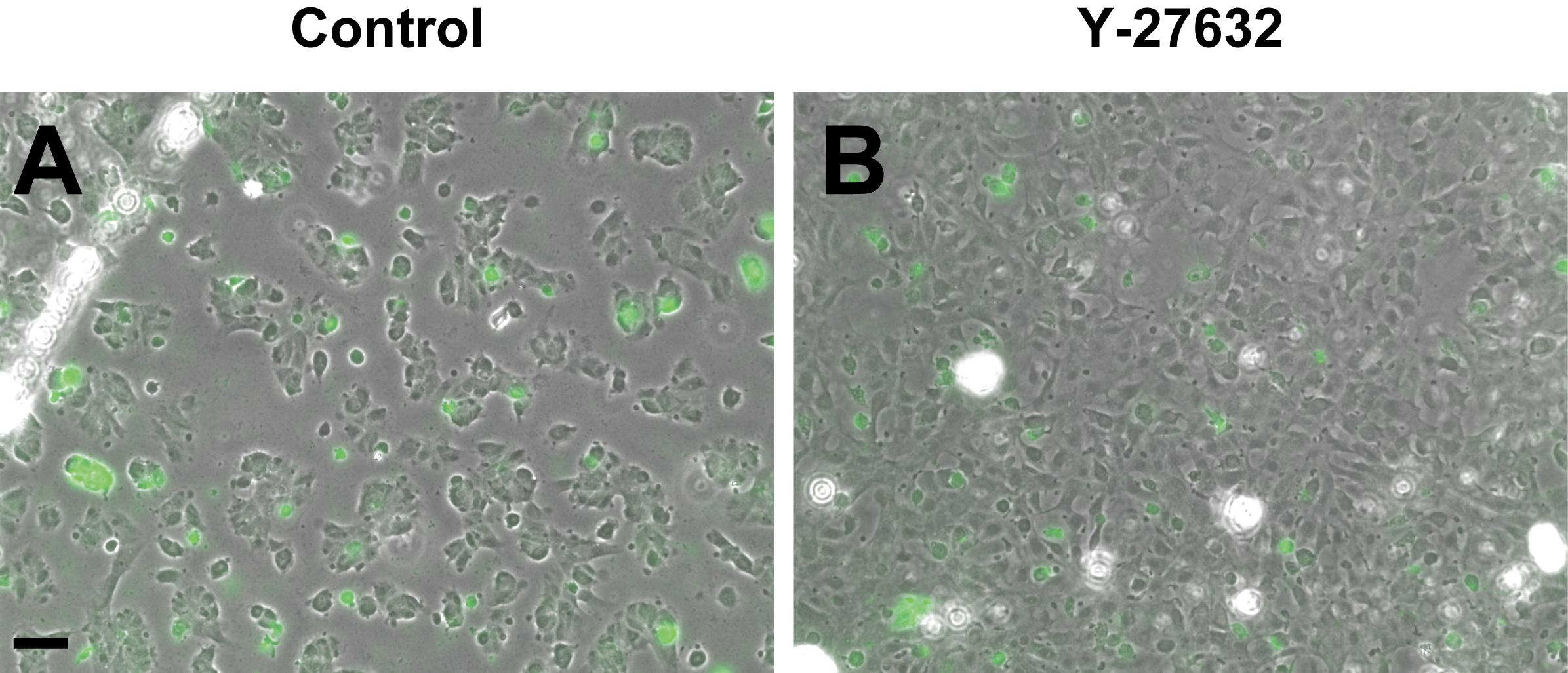

Supplement: S2 Fig — pCTBs were cultured with (B) and without (A) 10 μM Y-27632 for 12 h. Exposed phosphatidylserine, which occurs early in apoptosis, was detected with an FITC-conjugated annexin V (Annexin V assay kit (MBL; Nagoya, Japan)) in accordance with the manufacturer’s instructions. (TIF) [file pone.0177994.s002.tif]

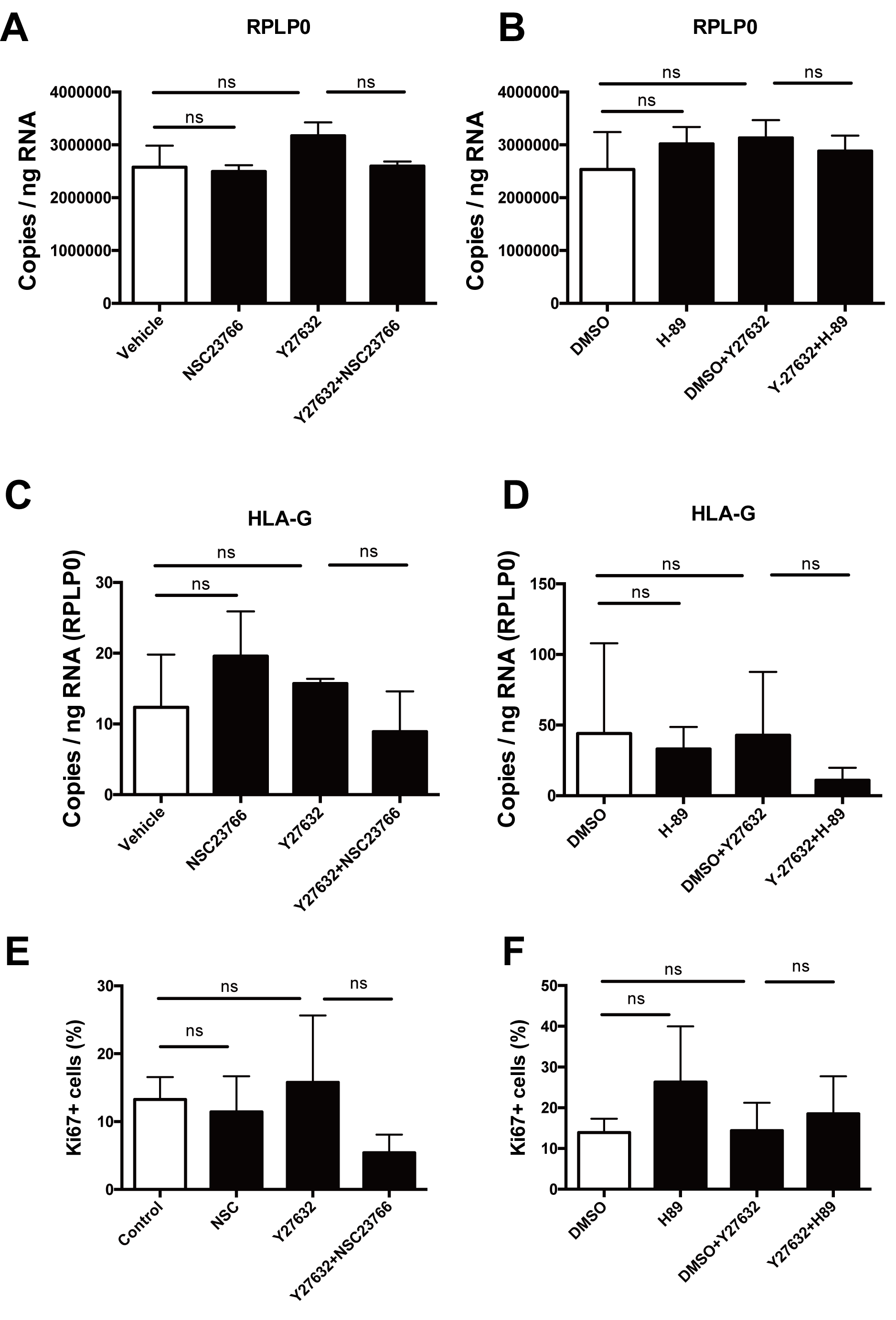

Supplement: S3 Fig — pCTBs were cultured with and without Y-27632, NSC-23766 (A, C, E) and H-89 (B, D, F) for 96 h. (A-D) Total RNA was extracted, and the mRNA expression levels for RPLP0 and HLA-G are shown. (E, F) Ki-67 of pCTBs was detected by immunofluorescence staining. The Ki-67–positive cell number was divided by the total nuclei number and shown as Ki-67+ cells. Each bar shows the mean of the results for 3 pCTBs from 3 different donors. Data are expressed as the mean ± SD. ns, not significant. (TIF) [file pone.0177994.s003.tif]

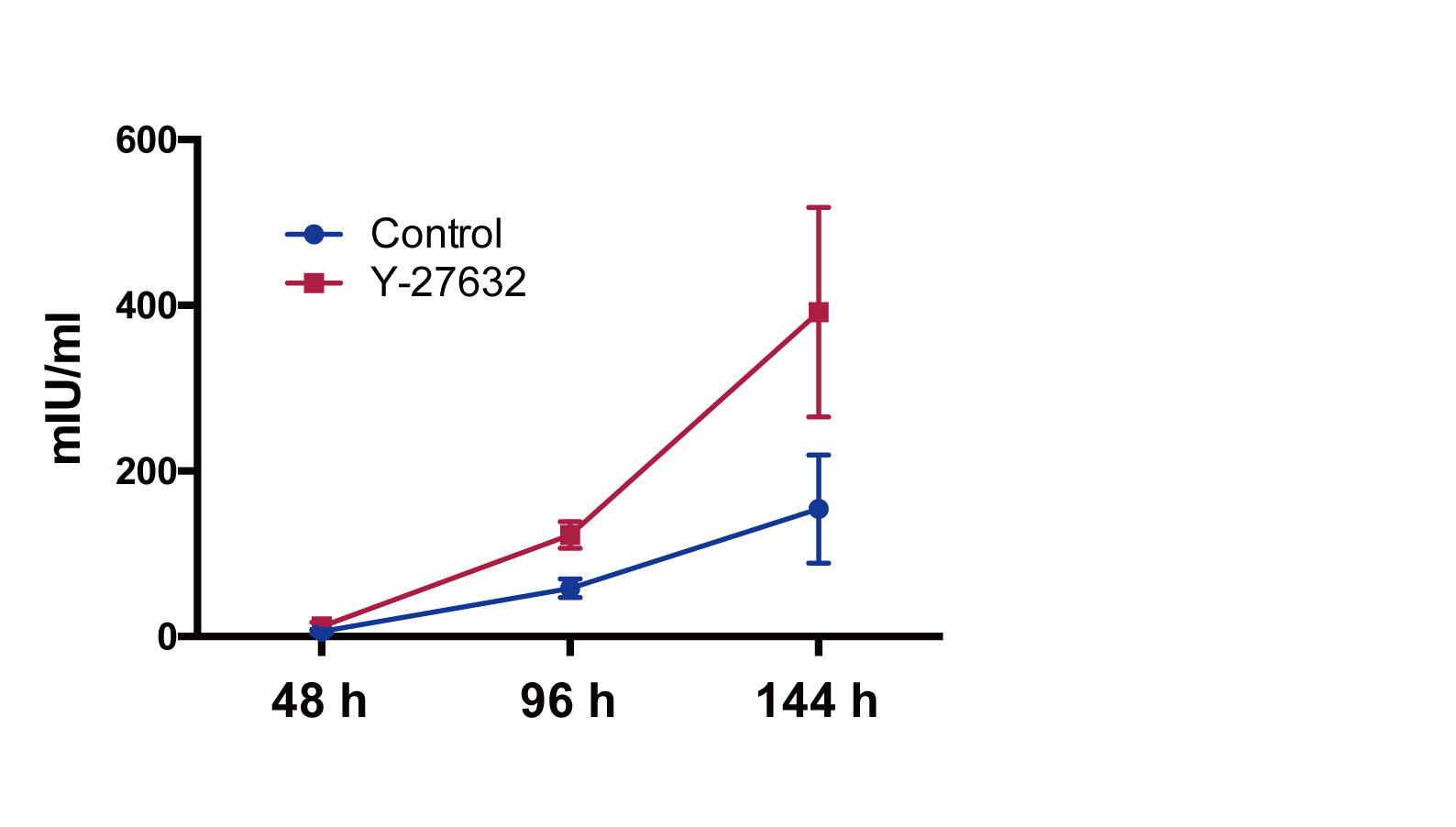

Supplement: S4 Fig — pCTBs were cultured with and without Y-27632 for 144 h. Culture supernatants were collected every 48 h, and the concentration of hCG-β was measured by ELISA. Each column shows the mean of the results for 3 pCTBs from 3 different donors. Data are expressed as the mean ± SD. (TIF) [file pone.0177994.s004.tif]

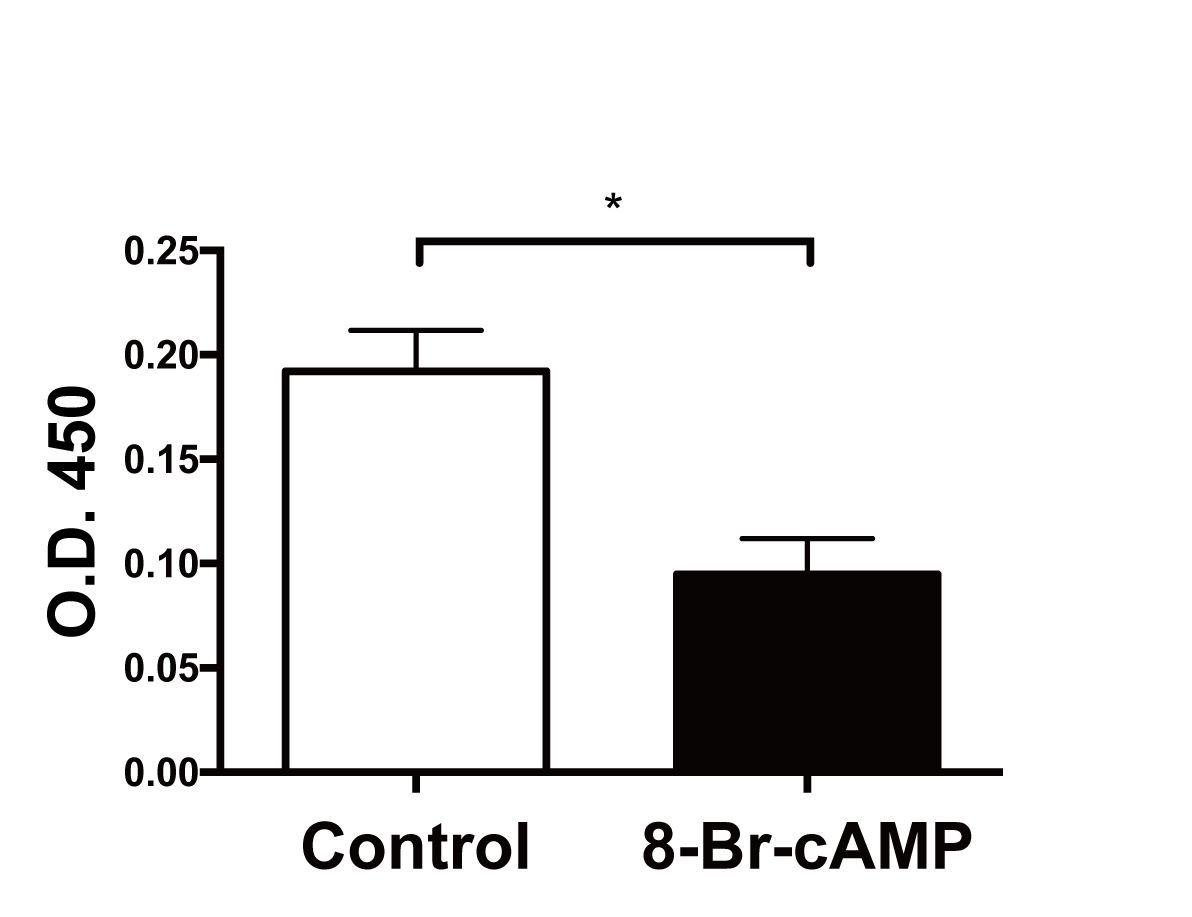

Supplement: S5 Fig — pCTBs were cultured with and without 8-Br-cAMP (10 μM) for 96 h. Cell viability was evaluated by WST-8 assay. The experiment was performed in quintuplicate. Data are expressed as the mean ± SD. *, P < 0.05. (TIF) [file pone.0177994.s005.tif]
